# The inter-relationship between depressed mood, functional decline and disability over a 10-year observational period within the Longitudinal Urban Cohort Ageing Study (LUCAS)

**DOI:** 10.1136/jech-2020-214168

**Published:** 2020-11-06

**Authors:** Ulrike Dapp, Christoph E Minder, Stefan Golgert, Björn Klugmann, Lilli Neumann, Wolfgang von Renteln-Kruse

**Affiliations:** 1 Scientific Department at the University of Hamburg, Albertinen-Haus Zentrum für Geriatrie und Gerontologie Medizinisch-Geriatrische Klinik, Hamburg, Germany; 2 Horten Zentrum, University of Zürich, Postfach Nord, CH 8091 Zürich, Zürich, Switzerland

**Keywords:** Functioning and disability, depression, longitudinal studies, geriatrics, ageing

## Abstract

**Background:**

The WHO defines ‘healthy ageing’ as ‘the process of developing and maintaining the functional ability’. Late-life depression and frailty compromise well-being and independence of older people. To date, there exists little research on the interaction of the dynamic processes of frailty and depression and only a few studies were longitudinal. Conclusions about the direction of effects remained uncertain.

**Methods:**

Data were obtained from each of the last six biyearly waves (2007–2017) of the Longitudinal Urban Cohort Ageing Study (LUCAS) in Hamburg, Germany, a prospective observational cohort study of manifold aspects of ageing. Screening of predictor and event variables: depressed mood: one question from the 5-item Mental Health Inventory Screening Test; frailty: LUCAS Functional Ability Index, status ‘frail’; disability: one question on need for human help with basic activities of daily living. Kaplan-Meier curves and Cox’s proportional hazards regression were used for time-to-event analyses with shifting baseline.

**Results:**

Sample size in 2007 was 2012, average age 76.2 years; ±6.5. Main results were as follows: (1) depression significantly increased the hazard of subsequent frailty (HR=1.581; 95% CI 1.257 to 1.988; p<0.001); (2) frailty significantly increased the hazard of subsequent depression (HR=2.324; 95% CI 1.703 to 3.172; p<0.001); (3) depression significantly increased the hazard of subsequent disability (HR=2.589; 95% CI 1.885 to 3.557; p<0.001) and (4) disability did not significantly increase the hazard of subsequent depression (HR=1.540; 95% CI 0.917 to 2.579; p=0.102).

**Conclusion:**

Our results suggest an interdependence of the processes of depression and frailty/disability rather than unidirectional dependencies. These observable processes may be representative of underlying unobservable profound life changes. Obviously, there is a need for early screening to initiate appropriate interventions.

## INTRODUCTION

The WHO definition of ‘healthy ageing’ is ‘the process of developing and maintaining the functional ability that enables well-being in older age’.^1^ Thus, a relevant public health issue is to prevent the development of disability.^1^ Frailty as characterised by an older person’s high vulnerability^2^ increases the risk of adverse outcomes including disability.^3^ Depression has received an interest as a disease which may interact with the frailty process.^4^ It has long been known that depression does worsen health problems and increases mortality in older people.^5 6^

The development of frailty was addressed in several theoretical frameworks. Bergman and colleagues^7^ consider physical and mental health components as frailty candidate components leading to adverse outcomes such as disability or death.^7^ A different publication^8^ hypothesised causal relationships between disability acquisition, mediators and future poor mental health.^8^ In contrast to these two approaches, Fillit and Butler^9^ hypothesised that incipient physical frailty is associated with a psychological state termed the ‘frailty identity crisis’.^9^ This stage of life is burdened with psychological challenges such as a sense of ‘becoming old’, regrets, sadness and depression. Thus, the ‘frailty identity crisis’ was seen as parallel or alternating processes of physical and mental deterioration.

Research on depression and frailty is difficult because both frailty and depression are dynamic processes.^4 10^ Nevertheless, relationships between depression and frailty - depressed mood and functional decline respectively - have been the subject of many cross-sectional and a few longitudinal studies. Recent systematic reviews and one meta-analysis of those studies^11–13^ found associations between depression and frailty. However, findings were contradictory regarding the direction of effect, with considerable variation in the definitions of frailty and depressive symptomatology used. As cross-sectional studies are not suitable to discriminate between the conceptual frames mentioned above, we compare our results to the four longitudinal cohort studies known to us.^14–17^

Three main features of our study differ from previous studies. First, it is based on data from a longitudinal cohort of 2012 persons with six questionnaires administered to each cohort member at 2-year intervals. Thus, we had recurrent information for each cohort member on both mental and functional health over 10 years.^18^ Second, our data set permitted the use of a measure of functioning^19^ derived from the concept of Fried and colleagues.^3^ Third, the six repeated observations made time-to-event analyses and the estimation of HRs possible.

### Research questions

Our aim was to investigate the mutual relationships between frailty, disability and depression/depressed mood. Therefore, our research questions were as follows:

1. Do persons reporting depressed mood have an increased risk of subsequent functional decline/frailty in contrast to persons who do not report depressed mood?
2. Do persons reporting functional decline/frailty have an increased risk of subsequent depressed mood over those not reporting functional decline?
3. Do persons reporting depressed mood have an increased risk of subsequent disability/need for human help with basic activities of daily living (BADL) over those not reporting depressed mood?
4. Do persons reporting disability/BADL dependency have an increased risk of subsequent depressed mood over those not reporting disability?

Our study is the first longitudinal study working with frailty phenotype criteria and distinct disability. Our longitudinal approach might permit discrimination between the conflicting concepts described above.^7–9^

## METHODS

### Study population

The data were obtained from the Longitudinal Urban Cohort Ageing Study (LUCAS) in Hamburg, Germany. LUCAS is a prospective observational cohort study to evaluate transitions from independence to frailty and disability^20^ within the geriatric functional continuum.^21^ There was no upper age cut-off in the LUCAS cohort. Due to high participation rates in all waves, extensive data on functional status, health behaviour and health outcomes as well as differential changes over time were available for analyses. Many factors from socio-demographic, medical, functional, behavioural and environmental domains were collected. Factors influencing these functional status transitions and sojourn times are of particular interest both for prevention and for healthcare services planning.

The data we used were from a suitable subset of data collected for the LUCAS cohort between 2000 and 2017. Initially (in 2000), general practitioners (GPs) from the entire metropolitan area Hamburg were invited to participate in the study (newsletter of the regional GP association). Twenty-one GPs working in solo practices agreed to participate and were recruited. These GPs were requested to provide complete lists of all their patients aged 60 years and older.^18^ For the present study, all participants who still could be contacted in 2007 and who were alive and willing to continue their participation were incorporated. The latest six biyearly LUCAS waves were used in our analysis. The flow chart in the online supplemental appendix figure S1 gives information on the numbers of participants in 2007/08 (n=2012) and dropouts over 10 years. The LUCAS study design was described in more detail elsewhere.^18^

10.1136/jech-2020-214168.supp1 Supplementary data

### Predictor and endpoint variables

The self-administered questions asked on depressed mood, functional decline and disability—subsequently used as predictor and event variables (response)—were phrased identically in all six LUCAS waves.

Depressed mood was assessed using one question from the 5-item Mental Health Inventory Screening Test, a validated questionnaire asking subjects about their mood over the last 4 weeks^22^: *‘*Have you felt so down in the dumps that nothing could cheer you up?’ with the possible responses ‘Yes’ or ‘No’. We used the term ‘depressed mood’, as operationalised above, and depression or depressive symptoms as synonyms.

Functional decline/frailty was assessed using the LUCAS Functional Ability Index (FAI),^19^ incorporating the five frailty phenotype risk factors ‘weight loss, slow gait, weakness, exhaustion, reduced physical activity’^3^ plus ‘instability/falls’ and six resources focusing on good endurance, frequent outside walking, moderate and strenuous sports or recreation, regular volunteer work and no limitation of activity due to fear of falling. The LUCAS FAI is distinct from BADL dependency.^20^ It incorporates Fried’s phenotype frailty criteria,^3^ but also functional resources which may help to compensate functional losses, that is, the term ‘frail’ used here is broader compared to the conventional view. The FAI discriminates between four functional classes (robust, postrobust, prefrail and frail). It predicted change in functional status, future need of nursing care and mortality. In this study, we concentrated on the class frail (3–6 frailty markers and 0–2 resources) because (a) the most frequent transition was to the next worse functional class, (b) a reversal of functional decline was least likely in persons with status frail and (c) the time span to need of nursing care (disability) was shortest in persons with status frail. Further details concerning the FAI were described elsewhere.^19^ We used the term ‘frailty’, as operationalised above, and functional decline as synonyms.

The frailty markers and the resources in the LUCAS FAI do correspond to higher performance levels than BADL. Therefore, disability was assessed using one question on self-perceived need of human help with BADL^23^: ‘Do you need help from someone else with any of the following? feeding yourself, getting to the toilet, dressing yourself, bathing yourself, moving from bed to chair or standing up’ with the possible responses *‘*Yes, I need help from someone else’ or ‘No, I do not need help from someone else.’ We used the term ‘BADL dependency’, as operationalised above, and disability as synonyms.

### Statistical analyses

Simple comparisons were done with χ² and Student’s t-tests. The main hypotheses were analysed using time-to-event data with Kaplan-Meier curves and Cox proportional hazard regressions. Our other data were categorical with two exceptions: age and body mass index (BMI). There were no missing values with age. BMI was categorised into four categories: underweight, normal weight, overweight and missing. All other data were categorical and to each we added a missing category. We estimated a parameter for each missing category, amounting to imputation by maximum likelihood estimation. The parameters for the missing class did adjust for the loss of precision due to missingness.

**Figure 1:**
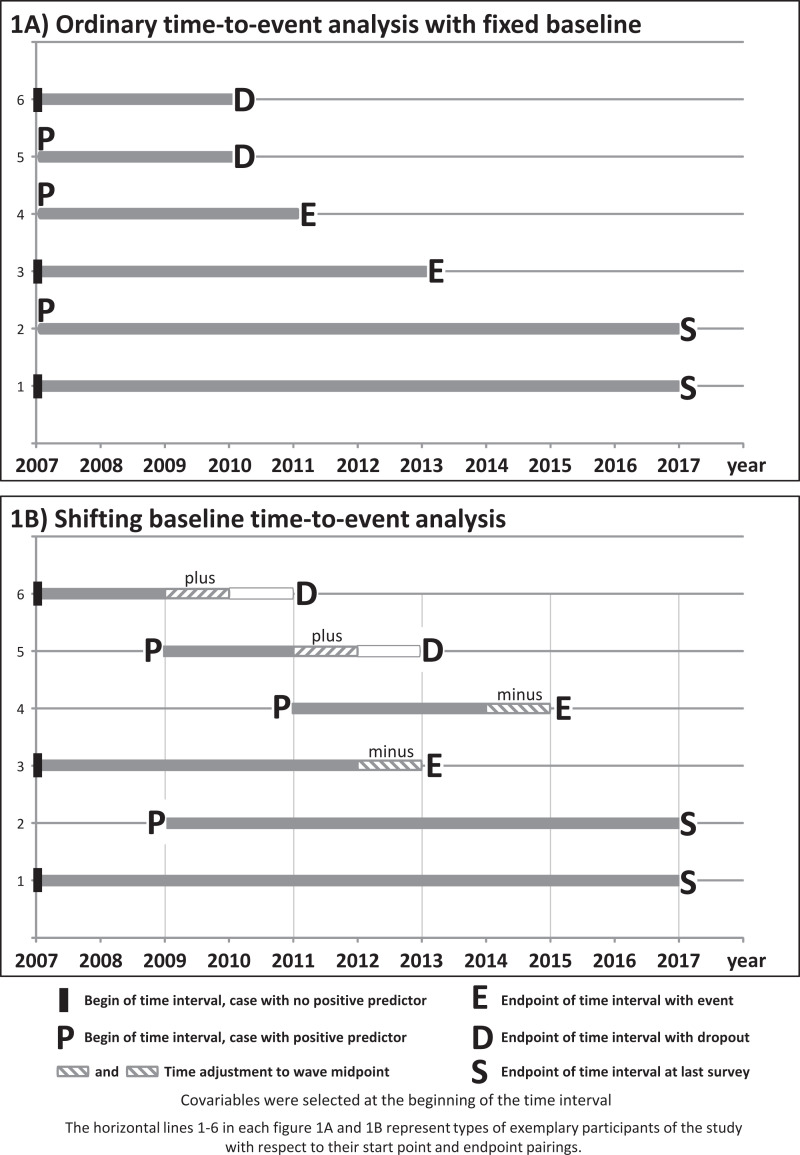
Ordinary and shifting baseline time-to-event analysis.

To perform our main data analyses concerning the effects of depressed mood on frailty or BADL dependency and vice versa, we used a modification of ordinary time-to-event analysis. As far as we know, such a modification has not been used before. Therefore, we describe it here in some detail. Ordinary time-to-event analysis is illustrated in figure 1A.

Our predictors depressed mood, functional decline (frailty) and disability (BADL dependency) were all binary; we gave them the generic term P signalling presence of the predictors (their absence with I). Similarly, events were termed E, dropouts D and time of last survey S. Thus, time intervals began either in 2007 or at the first presentation of P and ended either with E, D or S. Individuals who showed E already at the first wave by necessity were omitted. Three aspects were different in our data from ordinary time-to-event data (see figure 1B). First, our data were granular as LUCAS waves were performed biyearly. Second, we used the midpoint between two waves as time endpoints, that is, between the wave before occurrence of the event and the one when the event was reported (adjustments indicated by hatched lines, see figure 1B). That means the time spans were accurate only up to 1 year. For example, for persons responding at wave 2011, but no more at wave 2013 (ie, dropped out between 2011 and 2013), their time interval ended in year 2012 (marked as D for dropout). Third, in order to capture all first occurrences of our predictors, we used a shifting baseline. For those presenting P at least once, we started the time interval of observation at the first occurrence of P (predictor). We measured time spans from either study start in 2007 (in individuals without occurrence of the predictor) or from first occurrence of the predictor (in individuals with occurrence of the predictor). Baseline data, that is, the values of P, age, sex and education, were collected at the beginning of the time interval, that is, at varying time points (shifting baseline; figure 1B). The six examples (horizontal lines 1-6) illustrated in figure 1A, B depict types of participants of the study with respect to their start point and endpoint pairings, where ‘plus’ and ‘minus’ visualize time adjustments to wave midpoints to better estimate actual occurrences of endpoints.

To analyse these time-to-event data, we used Kaplan-Meier curves and Cox’s proportional hazards regression. In all Cox analyses, besides the predictor, we adjusted for age, gender and level of education. Education was classified using the International Standard Classification of Education.^24^ The predictors, endpoints and cofactors used are given in the Results section. We also did sensitivity analyses. For all analyses, we used Stata 15, for tabulations Excel and Word, for graphs Stata 15 and PowerPoint. P values <0.05 were considered as significant.

The use of personal data in the LUCAS study was agreed upon and was in accordance with the principles of the Declaration of Helsinki, the rules of the German Personal Data Protection Act and the Hamburg Data Protection Act. All personal data used were approved and approvals were updated periodically by the Ethics Committee of the General Medical Council Hamburg (PV-2856) in 2007, 2011, 2015 and 2017.

## RESULTS

Factors characterising the population are listed in table 1. At baseline (wave 2007), 2012 LUCAS participants filled in the self-administered questionnaire. Mean age was 76.2 years (±6.5) and 63.1% were women. Higher education (A-level or secondary school) was reported by 39.9% of the participants. In wave 2007, the prevalence of individuals with depressed mood was 11.7%, of those with functional decline (LUCAS FAI class frail) 25.6% and 6.8% had a disability (BADL dependency).

**Table 1:** Characteristics of participants at study start wave 2007 and at last wave 2017

Characteristics (self-reported)	Expression	Participants LUCAS wave 2007%	Participants LUCAS wave 2017%
Group size	Number	2012	776
Women		63.1	61.9
Age at survey in years	Mean±SD	76.2±6.5	82.8±4.6
Education*	At least 10 years of school education (ISCED level 3 or 4)	39.9	42.7
Self-reported health†	Fair or poor	39.0	43.0
Heart disease‡	Yes	22.3	22.0
Diabetes§	Yes	16.1	16.4
Neurological disease¶	Yes	5.7	6.5
Pain**	Yes	40.4	49.1
Urinary incontinence††	Yes	33.9	45.5
Depressed mood‡‡,§§	Yes	11.7	13.5
BADL¶¶,***	Restricted	6.8	8.1
Functional competence according to LUCAS Functional Ability Index†††	Robust Postrobust Prefrail Frail‡‡‡	52.2 10.9 10.4 25.6	44.1 11.7 7.9 36.3
Ride a bike§§§	Yes	45.7	31.2
Access to parks/green space¶¶¶	5 or more	62.6	65.6
Body mass index	Overweight (≥27)	37.6	36.8

*What type is your highest education degree you completed in school? highest degree (13 years of school education=ISCED level 4); medium degree (10–12 years of school education=ISCED level 3); basic degree (9 years of school education=ISCED level 2); degree in home economics (9 years of school education=ISCED level 2); no graduation (ISCED level 0); other (ISCED level 2); education was harmonised with the International Standard Classification of Education, ISCED.^24^

†In general would you say your health is: (very good; good; fair; poor).

‡Have you ever had angina pectoris or coronary heart disease or a heart attack? (no; yes).

§Do you have diabetes? (no; yes).

¶Do you suffer from a disease of the nervous system that causes uncontrolled tremor (eg, Parkinson’s disease) or a paralysis (eg, caused by a stroke) or a neurological disorder that causes disturbed coordination (eg, multiple sclerosis)? (no; yes).

**Do you have pain that never completely goes away? (no; yes).

††Are you having problems holding your urine? (no; yes).

‡‡During the last month have you felt so down in the dumps that nothing could cheer you up? (no; yes).

§§Predictor and event variable ‘depressed mood’.

¶¶Do you need help from someone else with any of the following? feeding yourself, getting to the toilet, dressing yourself, bathing yourself, moving from bed to chair or standing up (no, I do not need help from someone else; yes, I need help from someone else).

***Predictor and event variable ‘disability’/‘BADL dependency’.

†††Classification according to LUCAS functional index,^19^ n=17 (0.8%) were not classified due to missing marker questions in 2007.

‡‡‡Class FRAIL (3–6 frailty markers and 0–2 resources) as predictor and event variable ‘functional decline’/‘frailty’.^19^

§§§ Do you cycle? (no, never learnt; no, given up; yes, sometimes; yes, regularly at least once a week).

¶¶¶Number of different green spaces/parks which can be accessed (entrance) on footpath/street within 1250 meters distance from home address.

BADL, basic activities of daily living; LUCAS, Longitudinal Urban Cohort Ageing Study.

**Table 2:** Number of cohort members showing relevant predictor and event combinations (the total is smaller than n=2012, as some participants did not contribute observation time because the event already occurred at the first wave; for corresponding graphs and HRs, see figure 2A–D)

Cases	Frequency*	Percent
(a) Predictor: DeprMood −> Event: FunctDecline (n=1444)		
DeprMood and later FunctDecline	90†	6.2
DeprMood but no later FunctDecline	140‡	9.7
No DeprMood but later FunctDecline	431§	29.9
No DeprMood and no later FunctDecline	783¶	54.2
(b) Predictor: FunctDecline −> Event: DeprMood (n=1707)		
FunctDecline and later DeprMood	116	6.8
FunctDecline but no later DeprMood	705	41.3
No FunctDecline but later DeprMood	108	6.3
No FunctDecline and no later DeprMood	778	45.6
(c) Predictor: DeprMood −> Event: Disability (n=1834)		
DeprMood and later Disability	60	3.3
DeprMood but no later Disability	344	18.8
No DeprMood but later Disability	112	6.1
No DeprMood and no later Disability	1318	71.9
(d) Predictor: Disability −> Event: DeprMood (n=1709)		
Disability and later DeprMood	18	1.1
Disability but no later DeprMood	184	10.8
No Disability but later DeprMood	208	12.2
No Disability and no later DeprMood	1299	76.0

*Interpretation of the frequencies analogous to footnotes of table 2a.

†These 90 individuals reported at one wave being in a depressed mood (DeprMood) and at a later wave functional decline (FunctDecline).

‡These 140 individuals reported at one wave being in a depressed mood (DeprMood) and at later waves never showed functional decline (FunctDecline).

§These 431 individuals reported at no wave being in a depressed mood (DeprMood) before they experienced functional decline (FunctDecline).

¶These 783 individuals reported at no wave being in a depressed mood (DeprMood) nor did they experience functional decline (FunctDecline).

Ten years later, 776 remaining participants had provided their answers to all six LUCAS waves between 2007 and 2017 (for dropouts, see online supplemental appendix figure S1). In 2017, the individuals had a higher mean age (82.8±4.6). The other characteristics were similar to those reported in 2007 with three exceptions. In 2017, more individuals reported having severe pain, urinary incontinence and frailty than in 2007 (table 1).

**Figure 2:**
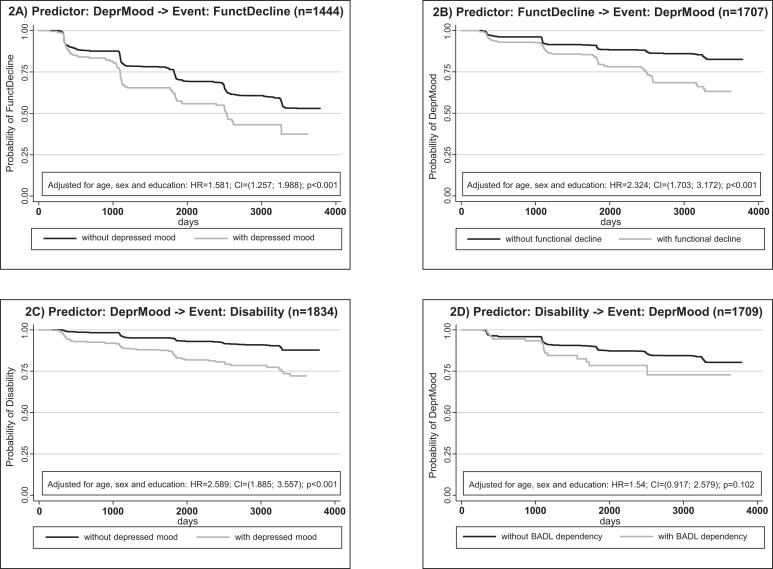
Time-to-event analysis; for frequencies of time courses, predictors and event combinations, see also table 2(a–d).

Figure 2 presents four Kaplan-Meier curves along with hazards ratios, CIs and p values derived from Cox regressions with the factors predictor (ie, either new depressed mood or new functional decline or new disability), age at time of predictor, sex and education. Figure 2A, B present the results on the relationship between depressed mood and functional decline. Depressed mood (figure 2A) significantly increased the hazard of subsequent functional decline (HR=1.581; 95% CI: 1.257 to 1.988; p<0.001). Functional decline (figure 2B) significantly increased the hazard of subsequent depressed mood (HR=2.324; 95% CI: 1.703 to 3.172; p<0.001). Figure 2C, D refer to the relationship between depressed mood and disability. Depressed mood (figure 2C) significantly increased the hazard of subsequent disability (HR=2.589; 95% CI: 1.885 to 3.557; p<0.001). Disability (figure 2D) did not significantly increase the hazard of subsequent depressed mood (HR=1.540; 95% CI: 0.917 to 2.579; p=0.102).

We did sensitivity analyses of all four models (predictors frailty or BADL dependency with event depressed mood, and predictor depressed mood with event frailty or BADL dependency), replacing the non-significant covariable education by each of the variables from table 1 in turn. None influenced the effects reported to an important degree.

Table 2 proved helpful for interpreting these results. First, for each analysis, the smallest and therefore crucial class was the one where both predictor and event occurred. Their numbers varied from 116/1707 (6.8%) (table 2b) to 18/1709 (1.1%) (table 2d).

**Figure 3:**
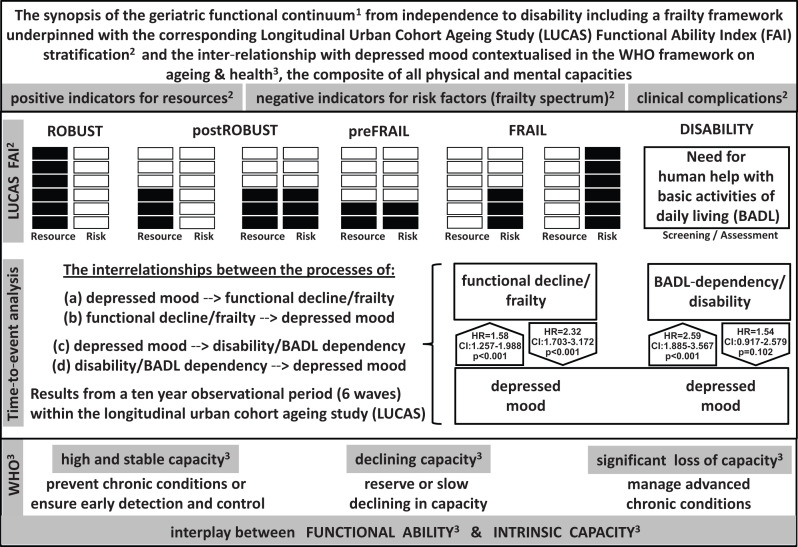
The inter-relationship between depressed mood, functional decline and disability embedded in a framework on ageing and health

^1^Modified on basis of theoretical models from Whitson *et al*^21^ and Bergman *et al*^7^. ^2^LUCAS Functional Ability Index (FAI): considering equally both, functional resources and risk factors distinct from disability.^19 3^A public health framework for healthy ageing—opportunities for action across the life course.^1^

Table 2 also showed that in each of the analyses, most cohort participants were neither disabled nor frail nor depressed. Hence, the vast majority of older people in our cohort were not affected—neither by frailty nor by disability or depressed mood.

## DISCUSSION

Our study was designed to investigate the dynamic processes linking frailty, disability and depressed mood in the LUCAS cohort of 2012 participants over 10 years in six biannual waves. We did a time-to-event analysis with shifting baseline as described above (figure 1B). In our analyses, depressed mood predicted subsequent occurrence of frailty and, even stronger, subsequent BADL dependency. Conversely, frailty predicted subsequent depressed mood but BADL dependency did not significantly predict later depressed mood. In all of these analyses, we adjusted for differences in gender, age and education.

The most pronounced finding, depressed mood predicting disability (HR 2.589, p<0.001; figure 2C) does fit the theoretical framework on frailty and ageing by Bergman and colleagues.^7^ They hypothesised that frailty is the result of a reduction in reserve capacity in multiple systems. Physical and mental components (depressive symptoms) are taken as frailty components, resulting in adverse outcomes such as disability.^7^

However, our results do not show a significant relationship between disability and future poor mental health (figure 2D) as hypothesised by Aitken and colleagues.^8^ A small sample size may have led to this non-significant result in figure 2D and table 2d. For all other analyses (see table 2a-c and figure 2A-C), the frequencies were sufficient (k>50). Although our results did not support Aitken and colleagues,^8^ they did not allow us to clearly reject their hypothesis either.

LUCAS partners doing practical geriatric work had observed that depressed mood diminished once disability had set in, participants in need of daily help had the challenge of adjusting to the new situation of being dependent.^25 26^ The present results together with those observations support the concept of the ‘frailty identity crisis’ by Fillit and Butler.^9^ Our results suggest that their concept may be a suitable framework to understand the inter-relationship between depressed mood and frailty/disability.

The result of frailty predicting depressed mood (figure 2B) was in accordance with results from previous longitudinal studies,^14–17^ although definitions and study designs varied. The three older studies did not investigate whether depression predicted frailty (figure 2A) or BADL dependency (figure 2C). Only Chang and colleagues^17^ studied both directions, as we did. They examined the co-occurrence of frailty (adapted Rockwood Frailty Index (RFI)) and depressive symptoms (CES-D) in 3–4 years of intervals of the Taiwan Longitudinal Study of Aging (TLSA; n=3352, mean age men 68.2 years, women 69.5 years) over a period of 18 years. Depression predicted subsequent frailty, and frailty predicted subsequent depression. To our knowledge, no other longitudinal study has investigated both directions. Thus, our findings are in accordance with theirs.

There are differences between the TLSA and the present study. First, Chang and colleagues^17^ used the RFI which is suitable for a clinical setting. In contrast, the frailty measure used here is derived from Fried’s frailty phenotype. Being based on self-reports, it is suitable for use in a community setting. Second, RFI is applied in persons who are also disabled. We clearly distinguished between disability and frailty by using distinct measurements.^2 27^ In contrast to Chang *et al,* ^19^ we looked separately at the relationship of both frailty and disability to depressed mood. We consider it important to distinguish between frailty and disability with regard to the demands of both, the individual and the healthcare services.

Both the results from TLSA and the present study favour inter-relationships between the processes of depressed mood, functional decline and disability as described in the ‘frailty identity crisis’.^9^

### Limitations and strengths

As other studies, we used self-reported data. Particularly regarding mental problems, this may be a drawback, although persons with cognitive impairment (equivalent to a Mini Mental Status score ≤24) were excluded at baseline,^18^ and we used a standard question from the Mental Health Inventory Screening.^22^ We had no information on antidepressive medication or psychiatric treatment aiding in estimating severity of symptoms or disease. As many others, our study did not allow to differentiate between depressive episodes and long-term chronic depression.^11–13^

Losses and dropouts are present in all longitudinal studies and cannot be avoided entirely. By keeping the dropout rate low in LUCAS,^18^ we did the best we could do under the circumstances to limit their influence.

Compared to other longitudinal studies, our study had a large number of six screenings as well as a long follow-up time (10 years). In addition, an innovative time-to-event analysis with shifting baseline permitted estimation of HRs (figure 1). Relying on the frailty phenotype and on a familiar measure of BADL dependency is the strength of our study. The FAI has been validated by geriatric/gerontological assessments in cohort subgroups,^25^ and the FAI was predictive of adverse health outcomes.^19^ LUCAS data were shown to be representative of the older population of Hamburg at LUCAS wave 1 (2001) wave 2 (2007), wave 3 (2009) and wave 4 (2011) with respect to demography and basic health parameters collected from representative health surveys and from the Hamburg Central Registry.^28^

### Community health perspective

Our results reflect an inter-relationship between depressed mood, functional decline and disability over a 10-year period. These findings may increase the awareness of the adverse consequences of the dynamic processes linking late-life depression, frailty and disability (occurring in varying sequence). Considering the public health perspective, we integrated the results of this study in figure 3.

Figure 3 is a synopsis of the geriatric functional continuum from independence to disability^21^ including a frailty framework,^7^ the corresponding stratification into the robust, postrobust, prefrail or the frail subpopulation according to the FAI^19^ considering both risk factors distinct from disability^3^ and functional resources.^29^ Finally, we contextualised our results in the WHO framework on ageing and health based on intrinsic capacity as defined by the composite of all physical and mental capacities,^1^ which is a construct related to frailty.^30^

There are many risks of functional decline at higher ages including depression.^31^ These risks open up opportunities for health-promoting interventions by strengthening resources.^19 32^ The WHO report on ‘Ageing & Health’ identifies many entry points for multidimensional action.^33^ A survey from the MINDMAP consortium presents rich material on strategies and programmes for strengthening physical and mental capacities in older individuals and their dynamic interactions in an urban environment.^34^

Our results have implications for planning and maintaining healthy urban environments.^35 36^ Easily accessible and sensually stimulating urban environments such as well-maintained and equipped walkways and city parks with many visitors, recreational activities, water surfaces and bird life may help to maintain physical and mental resources by stimulating older people to get out of houses to walk, meet, chat and enjoy life.^37 38^

## CONCLUSIONS

Our results provide evidence for dynamic long-term interdependence between depressed mood, frailty and disability. In those affected by depressed mood and/or manifest functional decline, these aspects appear to be mutually linked. However, the vast majority of our urban cohort participants aged 67 years and older never reported depressed mood or symptoms of frailty or disability within the study period of 10 years.

An older person experiencing significant loss of mental and/or physical capacities in daily life has three options: (a) to be distressed, (b) to adapt or (c) to limit functional losses. Adaptation can be achieved by appropriate management and by removal of barriers to participation. Functional losses may be prevented by health-promoting activities targeting older people with still high and stable physical and mental capacities. For this purpose, a broad variety of interventions to encourage healthy behaviours and to provide healthy environments are available.^21 34^ The challenge is to provide those to the suitable subgroup (figure 3). Our study supports the view that regular screening for depressed mood and incipient functional decline may help to initiate early and appropriate interventions.^39^

**What is already known on this subject**

- Systematic reviews and one meta-analysis revealed associations between frailty and depressive symptoms in older people.
- Most studies were cross-sectional.
- Three out of the four longitudinal cohort studies found did not investigate bidirectional relationships between frailty and depression.
- Information about changes over longer time periods is rare.

**What this study adds**

- With six biyearly observations over 10 years of the Longitudinal Urban Cohort Ageing Study (LUCAS), using time-to-event analyses with shifting baseline, we found solid evidence for an interacting process between depressed mood and functional decline, and depressed mood and disability.
- Our study results provide a deeper understanding of the processes of becoming depressed, frail and disabled.
- Results and methods suggest community-based interventions on both the individual and community levels.
- Both study and methods used were developed for community-dwelling senior citizens and are well suited to community investigations.
