# Supplementary figures and images for: The inter-relationship between depressed mood, functional decline and disability over a 10-year observational period within the Longitudinal Urban Cohort Ageing Study (LUCAS)
Source: J Epidemiol Community Health. 2020 Nov 6;75(5):450–7. doi: 10.1136/jech-2020-214168 (PMC8053334; doi:10.1136/jech-2020-214168)

**Figure S1: Flowchart six bi-yearly LUCAS waves (2007-2017)**

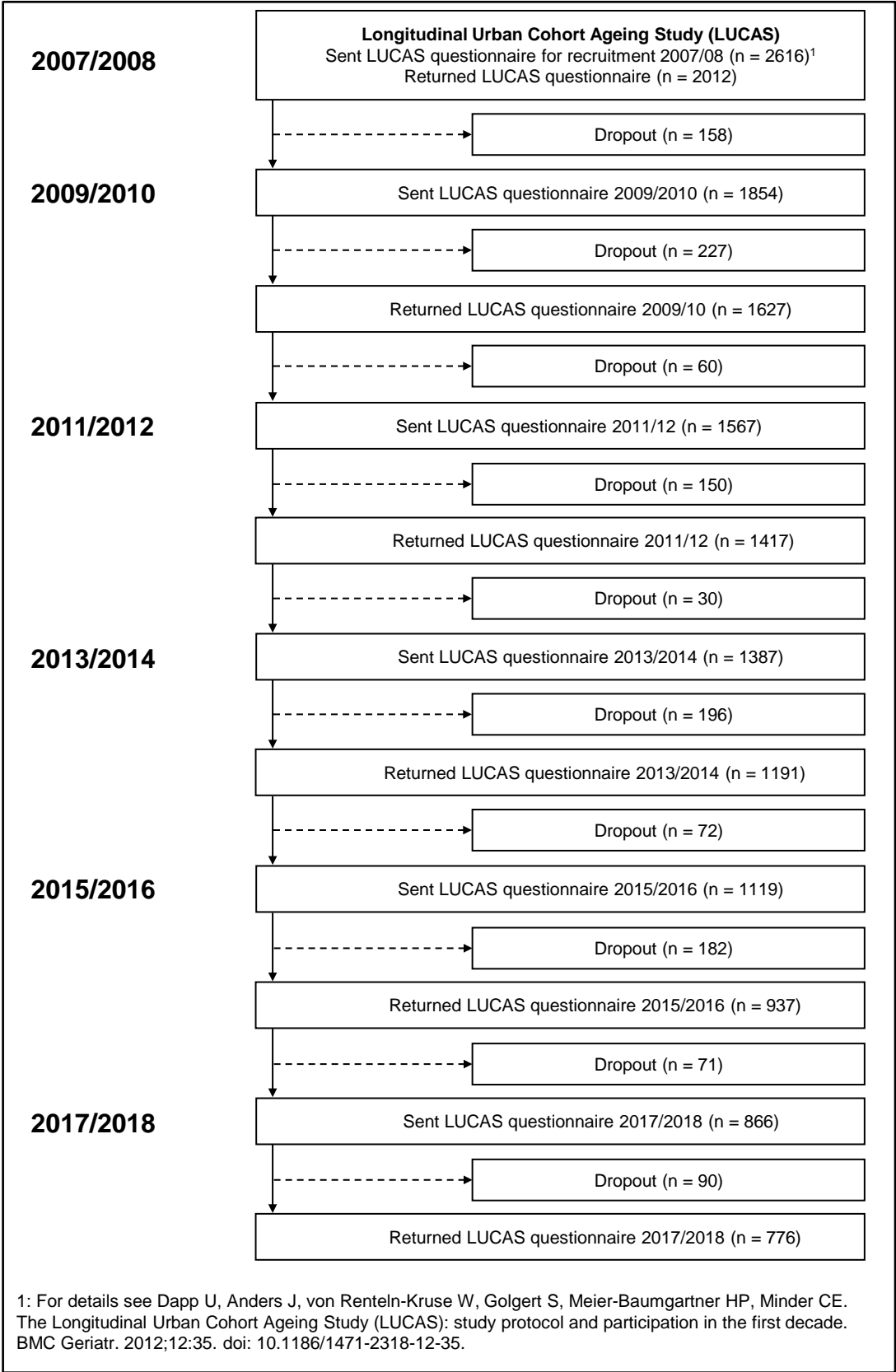

Supplement: Supplementary data [file jech-2020-214168supp001.pdf]
